# Human IL-35 Inhibits the Bioactivity of IL-12 and Its Interaction with IL-12Rβ2

**DOI:** 10.4049/immunohorizons.2300039

**Published:** 2023-06-08

**Authors:** Najmus S. Mahfooz, Marlena R. Merling, Tiffany A. Claeys, Jack W. Dowling, Adriana Forero, Richard T. Robinson

**Affiliations:** Department of Microbial Infection and Immunity, The Ohio State University, Columbus, OH; Department of Microbial Infection and Immunity, The Ohio State University, Columbus, OH; Department of Microbial Infection and Immunity, The Ohio State University, Columbus, OH; Department of Microbial Infection and Immunity, The Ohio State University, Columbus, OH; Department of Microbial Infection and Immunity, The Ohio State University, Columbus, OH; Department of Microbial Infection and Immunity, The Ohio State University, Columbus, OH

## Abstract

IL-35 is an immunosuppressive cytokine with roles in cancer, autoimmunity, and infectious disease. In the conventional model of IL-35 biology, the p35 and Ebi3 domains of this cytokine interact with IL-12Rβ2 and gp130, respectively, on the cell surface of regulatory T and regulatory B cells, triggering their suppression of Th cell activity. Here we use a human IL-12 bioactivity reporter cell line, protein binding assays, and primary human Th cells to demonstrate an additional mechanism by which IL-35 suppresses Th cell activity, wherein IL-35 directly inhibits the association of IL-12 with its surface receptor IL-12Rβ2 and downstream IL-12–dependent activities. IL-12 binding to the surface receptor IL-12Rβ1 was unaffected by IL-35. These data demonstrate that in addition to acting via regulatory T and regulatory B cells, human IL-35 can also directly suppress IL-12 bioactivity and its interaction with IL-12Rβ2.

## Introduction

IL-35 is an immunosuppressive cytokine that affects the progression of multiple diseases in mouse model systems, including allergy (1), autoimmune encephalomyelitis (2), inflammatory bowel disease (IBD) (3, 4), and cancer (5). Human IL-35 is expressed in several cancers, including lung cancer (6, 7), colorectal cancer (8, 9), and acute myeloid leukemia (10, 11), where it is believed to suppress anticancer immune responses. IL-35 has also been implicated in progression of the infectious disease tuberculosis (TB) because high IL-35 levels in the circulation (12) and pleural cavity (13) coincide with active forms of TB, which are more lethal than inactive or latent TB. Given the wide range of diseases influenced by IL-35, it is important to understand the mechanisms through which this cytokine mediates its immunosuppressive effects.

IL-35 is a member of the IL-12 family of heterodimeric cytokines, the founding member of which is IL-12 (14). IL-12 comprises two protein subunits—p35 and p40—that are linked via a disulfide bond and secreted by APCs in secondary lymphoid organs and inflamed tissues. IL-12R comprises two nonlinked proteins, IL-12Rβ2 (which associates with p35) and IL-12Rβ1 (which associates with p40), which, when engaged by IL-12, signal via intracellular STAT4 to elicit transcription of multiple proinflammatory genes, including that which encodes IFN-γ (*IFNG*). IL-35 is similar to IL-12 insomuch as it, too, contains a p35 subunit; where it differs, however, is that its p35 subunit is instead linked to Ebi3 (not p40). In the conventional model of IL-35–dependent immunosuppression, IL-35 signals through an IL-35R on the surface of T and B cells, directing them to become more like T regulatory (Treg) and B regulatory (Breg) cells, respectively, which in turn suppress the activity of numerous immune lineages, including Th1 cells (2, 4, 14). This model has been validated in large part using mice that lack these cellular intermediates (e.g., Treg and Breg cells) and/or are genetically deficient in individual components of the IL-35R complex, which comprises IL-12Rβ2 (which associates with p35) and gp130 (which associates with Ebi3). In the mouse system, IL-12Rβ2 homodimers and gp130 homodimers also signal in response to IL-35 (15).

We do not dispute the above conventional model; nevertheless, we set out to test if the immunosuppressive properties of IL-35 could also stem from competition with IL-12 for IL-12Rβ2 binding, because IL-35 and IL-12 each contain a p35 subunit, and IL-12Rβ2 is the principal signaling component of IL-12R (16). Our motivations for undertaking this study are 3-fold. (1) Because human and mouse IL-12 family members diverge significantly at the primary amino acid sequence level (17), as well as at the activity level (human IL-12 does not act on mouse cells, whereas mouse IL-12 acts on both human and mouse cells [18]), it is possible that human IL-35 may suppress human Th cells via mechanisms not observed in mice. (2) We sought to ascertain if human IL-35–dependent suppression could be observed in the absence of cellular intermediates such as Treg and Breg cells, because these lineages may be absent from tissues or cancers that may otherwise respond to IL-12–based immunotherapies. (3) Because IL-12 and IL-35 are potent regulators of multiple immunological processes, it is important to understand all of their overlapping effects on one another and downstream consequences in human systems.

## Materials and Methods

### IL-12 bioactivity reporter cells

The HEK-Blue IL-12 reporter cell line was purchased from InvivoGen (San Diego, CA) and cultured per the manufacturer’s instructions. This line comprises HEK293T cells that stably express human IL-12Rβ1, human IL-12Rβ2, and human STAT4, as well as a phospho-STAT4 (pSTAT4) responsive, secreted alkaline phosphatase (SEAP) reporter construct. Cells were maintained in culture media consisting of DMEM, 4.5 g/L glucose, 2 mM l-glutamine, 10% heat-inactivated FBS, penicillin-streptomycin (100 U/ml–100 μg/ml), and selection antibiotics (100 μg/ml Normocin). No pH indicator was added to the DMEM so that SEAP measurements (a colorimetric assay) were as sensitive as possible.

### IL-12, IL-23, and IL-35 response assay

Human IL-12 (PeproTech, Rocky Hill, NJ; 200-12H), IL-23 (PeproTech, 200-23), and IL-35 (PeproTech, 200-37) were reconstituted in sterile DMEM. To measure reporter cells’ response to IL-12 in the presence or absence of IL-35, ∼50,000 cells were added to each well of a 96-well plate and allowed to settle overnight (37°C, 5% CO_2_). The next morning, 20 μl IL-12 and/or IL-35 was added at the indicated final concentrations in triplicate (i.e., three biological replicates). IL-23 alone at the same concentrations was included as a control to determine the IL-12 specificity reporter cells. Cells were then incubated for 12 h (37°C, 5% CO_2_), after which the cell supernatants were used to measure SEAP levels via the QUANTI-Blue solution method (InvivoGen). Absorbance readings were taken (wavelength 630 nm), and dose–response curves were plotted using GraphPad Prism (version 8.0). Each IL-12 ± IL-35 dose–response assay shown was repeated at least three times.

### IL-12 and IL-35 immunofluorescence

IL-12 reporter cells were grown on tissue culture–treated coverslips until they were ∼60% confluent, at which point they were washed with prewarmed sterile PBS, and the media were replaced with fresh, prewarmed culture media (minus antibiotics), followed by 1-h incubation (37°C, 5% CO_2_). Cytokines were then added (either IL-12 alone, IL-35 alone, or IL-12 and IL-35 together) at a final concentration of 150 ng/ml. Cells were incubated for 10 min (37°C, 5% CO_2_) and then immediately fixed with an ice-cold methanol:acetone mixture (1:1) for 10 min. Fixed cells were blocked with 1% BSA/0.1% Tween-20 for 1 h and labeled with the following primary Abs overnight at 4°C: 1 µg/ml goat anti-human IL-12 (PeproTech, 500-P154G) and/or 1 µg/ml mouse anti-human IL-35 (BioSource, MBS2090569). Validation data demonstrating the specificity of anti-human IL-35 are provided in Supplemental Fig. 1. Cells were then washed and stained with the following secondary Abs for 1 h at room temperature: donkey anti-goat IgG (conjugate Alexa Fluor 647) and donkey anti-mouse IgG (conjugate Alexa Fluor 488). Each secondary Ab was used at a final concentration of 0.5 µg/ml in PBS with 1% BSA/0.1% Tween-20. Cell nuclei were stained with DAPI (final concentration 0.1 µg/ml) and washed three times with sterile PBS prior to imaging. Stained cells were visualized and analyzed using an Olympus FV1000 confocal microscope and ImageJ image analysis software.

### Colocalization analysis

To evaluate the overlap of IL-12 and IL-35 immunofluorescence channels of IL-12/IL-35-treated reporter cells, we used pixel intensity correlation measurements based on Pearson coefficient, using the JACoP (BIOP version) plugin in Fiji (19). Before analysis, background subtraction was performed for each channel using the rolling ball radius, which is the average pixel length size of cells in the image field of view. Images were thresholded using manual methods. Images were analyzed for Pearson’s correlation and a two-dimensional intensity fluorogram.

### Immunoprecipitation studies

Approximately 2–5 × 10^6^ IL-12 reporter cells were plated in a culture flask and allowed to settle overnight. After 1 d, the cells were grown in serum-starved media (0.5% FBS) for 12–16 h. This was followed by incubating cells with normal media (10% FBS) for 30 min. The cells were then stimulated with either IL-12 or IL-35 (150 ng/ml) for 10 min at 37°C, then washed with ice-cold 1× PBS, followed by cell lysis. Cells were lysed with lysis buffer (50 mM Tris-HCl, 150 nM NaCl, 1% Triton X-100, protease inhibitor mixture by Roche) for 30 min on ice, and cell lysates were centrifuged at 13,000 rpm for 10 min to remove insoluble materials. The supernatant was collected, and protein concentration was estimated using a bicinchoninic acid assay (Pierce). Approximately 100 μg of cell lysate was incubated with 1 μg of goat anti–IL-12 polyclonal Ab (PeproTech, 500-P154PG) or mouse anti–IL-35 mAb (BioSource, MBS335048) overnight at 4°C. Ab–Ag complexes were eluted using the Capturem coimmunoprecipitation kit (Takara, 120519) following the manufacturer’s instructions. Eluted samples were run on a 4–20% polyacrylamide gradient gel (Bio-Rad Laboratories) and transferred to a nitrocellulose membrane at 50 V for 2 h. The blot membrane was probed with mouse anti–IL-12RB2 primary mAb (R&D Systems, MAB19591) followed by donkey anti-mouse IRDye 800CW fluorescence Ab (LI-COR Biosciences, 925-32212). All images were captured using an Odyssey DLx fluorescence imager (LI-COR Biosciences).

### Solid-phase IL-12Rβ2 and IL-12Rβ1 binding assay

To test if IL-35 competes with IL-12 for IL-12Rβ2 or IL-12Rβ1 binding, independent of additional coreceptors, we developed a solid-phase binding assay wherein individual wells of a 96-well plate (Corning, 9018) were coated overnight (4°C) with recombinant human IL-12Rβ2 (Creative Biomart, IL-12RB2-8483H) or human IL-12Rβ1 (Sino Biological, 11674-H08H), as reconstituted in sterile PBS (50 μl per well of a 5 μg/ml solution). After the plate was washed three times with sterile PBS containing 0.05% Tween-20, the plate was blocked with 1× PBS/0.05% BSA for 1 h at room temperature. After washes, 50 μl IL-12 and/or IL-35 was added at the indicated final concentrations in triplicate to eventually calculate mean and SD values. For other experiments, IL-12p40 monomer (PeproTech 200-12P40) was used instead of IL-12 at the same concentrations. The plate was incubated overnight (4°C), after which the unbound cytokine was removed by washing three times with 1× PBS containing 0.05% Tween-20, and bound cytokines were detected using anti–IL-12 (PeproTech, 500-P154G), anti–IL-35 (BioSource, MBS335048), or anti–IL-12p40 (eBioscience, 14-7127-81) at a final concentration of 0.5 μg/ml followed by HRP-conjugated secondary Abs (1:4000). HRP activity was detected using tetramethylbenzidine substrate and measured on a standard plate reader at 570 nm.

### Jurkat T cell stimulation assay

The human Jurkat T cell line (American Type Culture Collection clone E6-1) was cultured and maintained in RPMI culture media supplemented with 10% FBS. For stimulating cells in the presence or absence of IL-12 with or without IL-35, we first plated ∼500,000 cells per well in 12-well culture plates and incubated them overnight at 37°C/5% CO_2_. The next day, PMA (final concentration 10 ng/ml) and ionomycin (Iono; final concentration 1 μg/ml) were added to the media, as well as a fixed concentration of IL-12 and/or IL-35 (IL-12, 2.5 μg/ml; IL-35, 625 ng/ml). Cells were harvested 2 h later and lysed with TRIzol (Invitrogen). RNA purification was carried out using the Direct-Zol RNA extraction kit method (Zymo Research, Irvine, CA), and cDNA synthesis was carried out using the VILO Superscript cDNA synthesis kit method (Invitrogen). For real-time PCR, TaqMan IFN-γ and GAPDH primer/probe mixes were purchased from Life Technologies (Carlsbad, CA), and the assays were run on a Bio-Rad CFX thermal cycler. All data were analyzed using Microsoft Excel and GraphPad Prism version 8.0.

### T cell polarization assay

Naive Th cells were purified via magnetic depletion from cryopreserved PBMC preparations of healthy adult donors using the Naive CD4+ T Cell Isolation Kit II method (Miltenyi Biotec). Naive Th cells were washed, counted, resuspended in complete RPMI media (RPMI 1640 supplemented with 10% heat-inactivated FBS, 1 mM Na-Pyr, 20 mM HEPES, 50 μM 2-ME), and added to 6 individual wells of a 96-well tissue culture plate (10,000–72,000 cells per well). Prewashed Human T-Activator CD3/CD28 Dynabeads (Thermo Fisher) were added to each well at a bead-to-cell ratio of 1:1. To test naive Th cells’ capacity for Th1 differentiation in the presence of absence of IL-35, we added to each well one of the following cytokine mixtures (final concentrations are indicated in parentheses): a Th1 without IL-35 mixture of IL-2 (10 ng/ml), IL-12 (2 ng/ml), and anti-IL-4 (5 μg/ml) or a Th1 with IL-35 mixture comprising the same as above plus IL-35 (2 ng/ml). Th1 cultures were set up in triplicate (i.e., of the six wells containing a given donor’s naive Th cells, three were Th1 without IL-35 and three were Th1 with IL-35). Cytokines and Abs were purchased from PeproTech and BioLegend (San Diego, CA), respectively. Cultures were incubated in a humidified 5% CO_2_ incubator at 37°C for 1 wk. Supplemental IL-2 was added to every well every other day for the entire week (days 2, 4, and 6). At the end of the 1-wk culture, the cells in each well were collected and restimulated in activation media (10 ng/ml PMA, 1 μg/ml Iono in complete RPMI) for 6 h. Supernatants were collected for quantification of IFN-γ by ELISA analysis (BioLegend) as a readout for Th1 capacity.

### Graphing and statistics

Graphs were prepared using GraphPad Prism or Microsoft Excel; data were statistically analyzed using their bundled software. Statistical comparisons involving more than two experimental groups used ANOVA with follow-up column-to-column comparisons. All other statistical comparisons used the Student *t* test. Differences between groups were considered significant if *p* < 0.05 and are graphically indicated by an asterisk (*p < 0.05).

## Results

### Human IL-35 directly inhibits IL-12–elicited STAT4 responses

**FIGURE 1.**
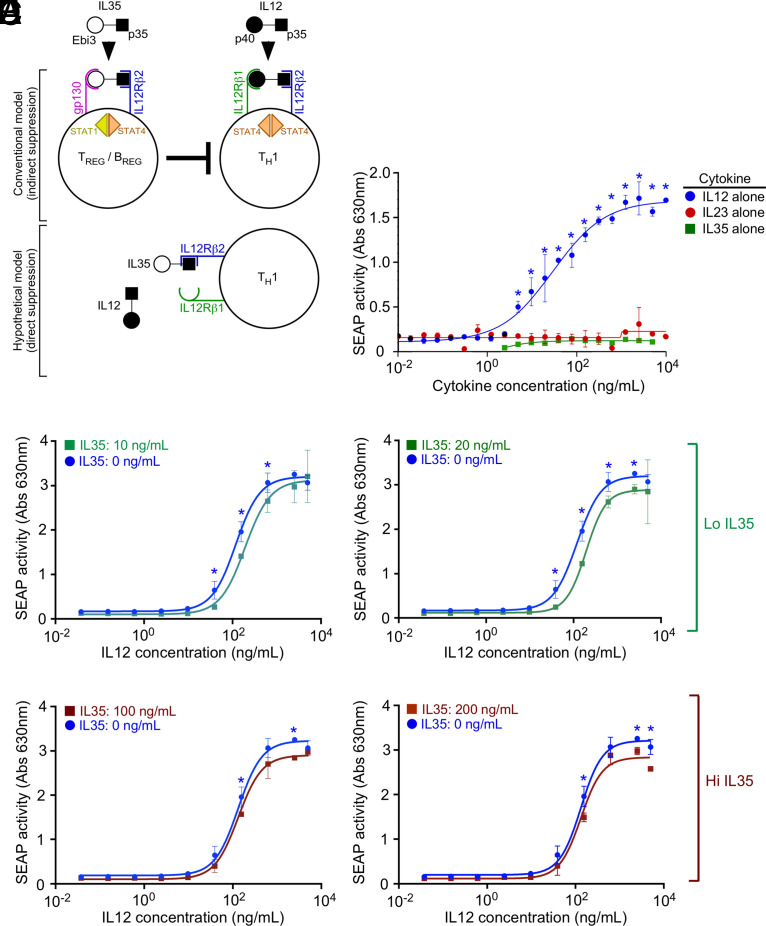
IL-35 directly inhibits IL-12 bioactivity. (**A**) Graphical depiction of the conventional model of IL-35–mediated immunosuppression (top panel) alongside the hypothetical model we aimed to test in this study (bottom panel). (**B**) An IL-12 bioactivity reporter cell line, which comprises HEK293T cells that constitutively express human IL-12Rβ1, human IL-12Rβ2, human STAT4, and a phospho-STAT4 (pSTAT4) responsive SEAP reporter construct, was cultured in increasing concentrations of either IL-12 alone (blue circles), IL-23 alone (red circles), or IL-35 alone (green squares), as added exogenously to culture media. SEAP activity in the reporter cell culture supernatant was measured 12 h later and used as a quantitative indicator of IL-12 bioactivity. (**C**–**F**) The experiment was repeated with IL-12 and IL-35 mixed together, wherein we exogenously added varying concentrations of IL-12 and one of four fixed IL-35 concentrations: (C) 10 ng/ml IL-35, (D) 20 ng/ml IL-35, (E) 100 ng/ml IL-35, and (F) 200 ng/ml IL-35. Cultures were performed in triplicate to generate the depicted mean ± SD values for each cytokine combination/concentration. Note that in (C–F), the same 0 ng/ml IL-35 control data are shown in all graphs for ease of comparison. These results are representative of five independent experiments. *p < 0.5 as determined by ANOVA.

IL-35 and IL-12 are heterodimers of two protein subunits (IL-35: p35 and Ebi3; IL-12: p35 and p40), the p35 subunit of which binds the transmembrane receptor IL-12Rβ2. We hypothesized that in addition to indirect suppression of Th cell IFN-γ secretion via IL-35R–dependent Treg and Breg cells (Fig. 1A, top panel), the immunosuppressive activities of IL-35 can also be due to IL-35 competition with IL-12 for IL-12Rβ2 binding (Fig. 1a, bottom panel). To test the hypothesis that IL-35 directly inhibits IL-12 bioactivity, we used a reporter cell line comprising HEK293T cells that constitutively express human IL-12Rβ1, IL-12Rβ2, and STAT4, as well as a phosphoSTAT4 (pSTAT4) responsive element driving the expression of SEAP. These cells are further described in *Materials and Methods* and hereafter are referred to simply as “IL-12 bioactivity reporter cells.” As anticipated, the addition of exogenous human IL-12 to these IL-12 bioactivity reporter cells caused a dose-dependent increase in SEAP levels within 1 d of addition and did not change in response to IL-23 (which uses IL-12Rβ1 and IL-23R, not IL-12Rβ2) (Fig. 1B). Less anticipated, however, because mouse IL-35 can use mouse IL-12Rβ2 homodimers to activate STAT4 (15), the addition of exogenous human IL-35 at the same concentrations did not affect SEAP levels (Fig. 1B). IL-23 also did not affect SEAP activity (Fig. 1B). We next assessed whether IL-35 affected IL-12–elicited SEAP when applied at the same time at low and high concentrations. Namely, exogenous IL-12 was added to reporter cells across a range of concentrations as before, except that IL-35 was also added simultaneously at two low concentrations (Fig. 1C, 10 ng/ml IL-35; Fig. 1D, 20 ng/ml IL-35) or two high concentrations (Fig. 1E, 100 ng/ml; Fig. 1F, 200 ng/ml). The results indicate that at low concentrations, exogenous IL-35 exhibited modest but nonetheless significant suppression of IL-12–elicited SEAP, as evidenced by a right shift of the IL-12 dose response in the presence of either the two low IL-35 concentrations (Fig. 1C, 1D). Curiously, this right shift was less prominent in the presence of either the two high IL-35 concentrations (Fig. 1E, 1F). Low IL-35 concentrations (IL-35^LO^) and high IL-35 concentrations (IL-35^HI^) both suppressed IL-12–elicited SEAP activity; unlike IL-35^LO^, however, IL-35^HI^ suppression was seen only at high IL-12 concentrations. Potential explanations for this observation are included in the *Discussion*.

### Human IL-35 interferes with IL-12 binding to the cell surface

**FIGURE 2.**
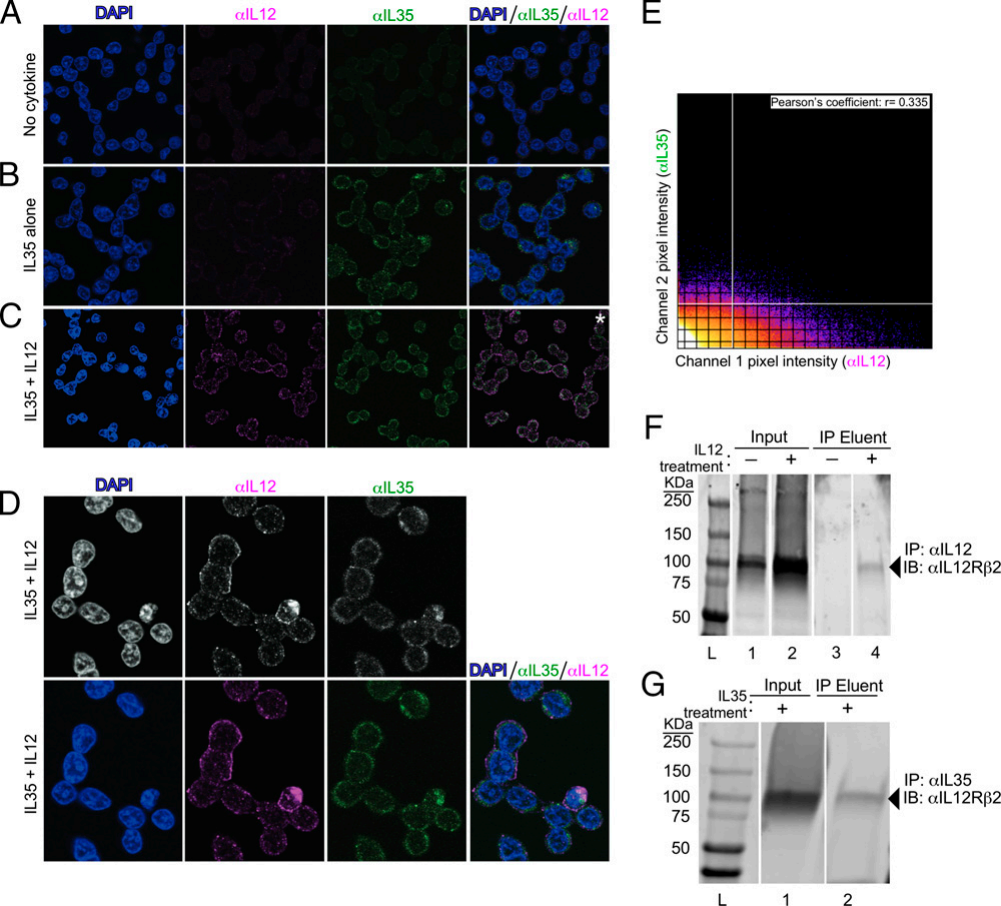
IL-12 and IL-35 associate with the cell surface in a nonoverlapping manner. To visualize whether IL-12 and IL-35 associate with the cell surface in an overlapping or nonoverlapping manner, the same reporter cell line described above was cultured in the presence of either (**A**) no cytokine, (**B**) IL-35 alone, or (**C**, **D**) IL-35 and IL-12 together. Within 10 min of cytokine addition, the cells were fixed and labeled with fluorophore-conjugated Abs specific to IL-12 (pink) and IL-35 (green), as well as DAPI (blue). Each row shows the immunofluorescence staining pattern for a given field of view across channels. Note that in (C), the DAPI channel is not shown in the far right overlay for the purpose of enhancing visualization of pink (IL-12) and green (IL-35) signals; however, the DAPI channel is shown in the (D) higher-magnification images of the same IL-35 and IL-12 cotreated cells, both (D, top panel) without color and (D, bottom panel) with color. These images are representative of four independent experiments. (**E**) To quantitatively assess the extent of IL-12 and IL-35 colocalization or the lack thereof, we used pixel colocalization analysis of the images shown in (D). Shown is the two-dimensional intensity histogram from the output of this analysis, with the inset showing the Pearson correlation value. (**F**, **G**) Reporter cells were treated with either (F) IL-12 or (G) IL-35 for 10 min, after which they were immediately washed and lysed for immunoprecipitation purposes. Lysates from cells that were either left untreated (−) or treated with cytokine (+) were mixed with either (F) anti–IL-12 Ab or (G) anti–IL-35 Ab and subsequently passed over protein A columns to enrich for proteins associating with these Ab–Ag complexes. Cell lysates (input) and immunoprecipitation eluent were run on SDS-PAGE gels, and blots were identically probed with anti–IL-12Rβ2, followed by appropriate secondary Abs for imaging purposes. These blots are representative of three independent experiments. Images in (A)–(C) and (D) were taken at original magnifications ×100 and ×400, respectively. The asterisk (*) indicates that the specified panel in (C) does not include the DAPI channel per the explanation provided above.

**FIGURE 3.**
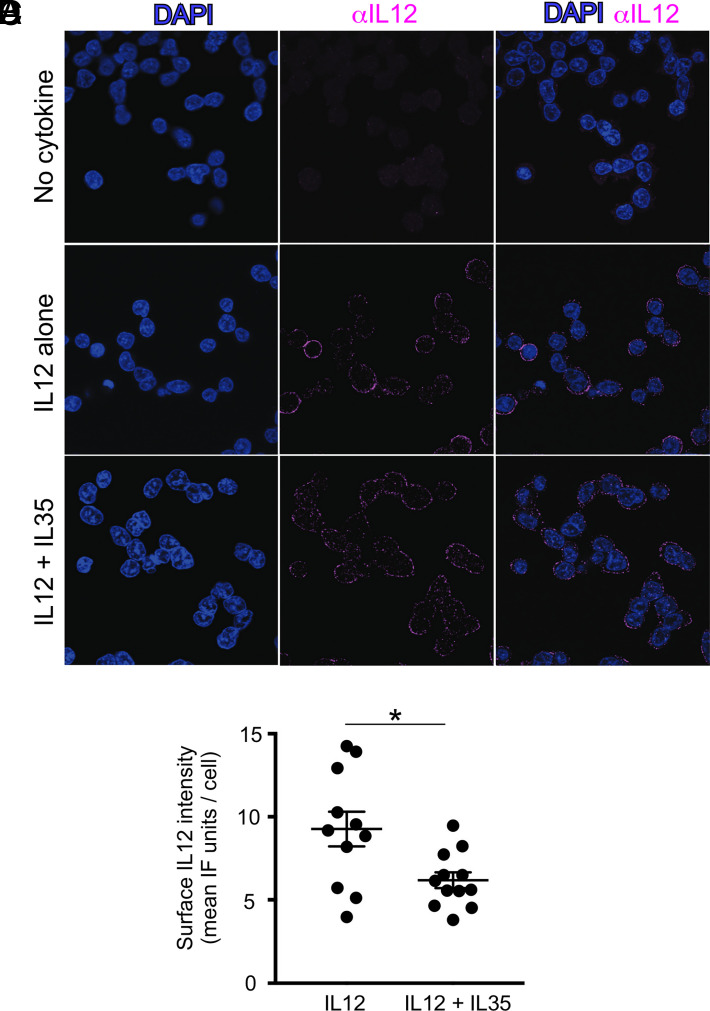
The association between IL-12 and the cell surface is weaker in the presence of IL-35. Reporter cells were cultured in the presence of either (**A**) no cytokine, (**B**) IL-12 alone, or (**C**) IL-12 and IL-35 together. Within 10 min of cytokine addition, the cells were fixed and labeled with a fluorophore-conjugated Ab specific to IL-12 (pink), as well as DAPI (blue). Each row shows the immunofluorescence (IF) staining pattern for a given field of view across channels. (**D**) Quantification of the IL-12 IF signal per individual cell in the absence or presence of IL-35. *p < 0.05 as determined by Student *t* test. These images and quantification results are representative of four independent experiments. Images in (A)–(C) were taken at original magnification ×100.

To determine if IL-35 inhibition of IL-12 signaling (Fig. 1) is due to inference with IL-12 binding the surface of reporter cells, we used immunofluorescence to visualize IL-35 and IL-12 on the surface of reporter cells within 10 min of their exogenous addition to the reporter cell media, either alone or in combination with one another. Compared with cells not treated with any cytokine (Fig. 2A), those treated with IL-35 reacted with anti–IL-35 in a manner consistent with cell surface staining (Fig. 2B). This suggests that the lack of pSTAT4 activity following addition of human IL-35 alone (Fig. 1A, open circles) is not due to its inability to bind the reporter cell surface. We confirmed that IL-35 physically associates with IL-12Rβ2 in this system via coimmunoprecipitating IL-12Rβ2 with anti–IL-35 Abs (within 15 min after IL-35 treatment), just as IL-12Rβ2 coimmunoprecipitated with anti–IL-12 Abs (within 15 min after IL-12 treatment) (Fig. 2F, 2G). When both IL-12 and IL-35 are added to reporter cells, the cell surface reacts with both anti–IL-12 and anti–IL-35 in a nonoverlapping manner (i.e., IL-12 and IL-35 do not colocalize) (Fig. 2C), consistent with IL-35 preventing IL-12 binding. Higher magnifications of cells cotreated with IL-12 and IL-35 also demonstrate that portions of the cell surface that bind anti–IL-12 (pink) do not overlap with portions that bind anti–IL-35 (green), as assessed visually (Fig. 2D) and quantitatively via pixel colocalization analysis methods (Fig. 2E, Supplemental Fig. 2). Cells treated with IL-35 alone do not bind anti–IL-12 (Fig. 2B). Regarding the effect of exogenous IL-35 on anti–IL-12 immunofluorescence staining intensity, as anticipated, compared with cells treated with no cytokine (Fig. 3B), the surface of those treated with IL-12 alone reacted with anti–IL-12 (Fig. 3C). Those treated with both IL-12 and IL-35 retained some reactivity with anti–IL-12, albeit diminished relative to those treated with IL-12 alone (Fig. 3D, 3E).

### Human IL-35 prevents IL-12 from binding IL-12Rβ2

**FIGURE 4.**
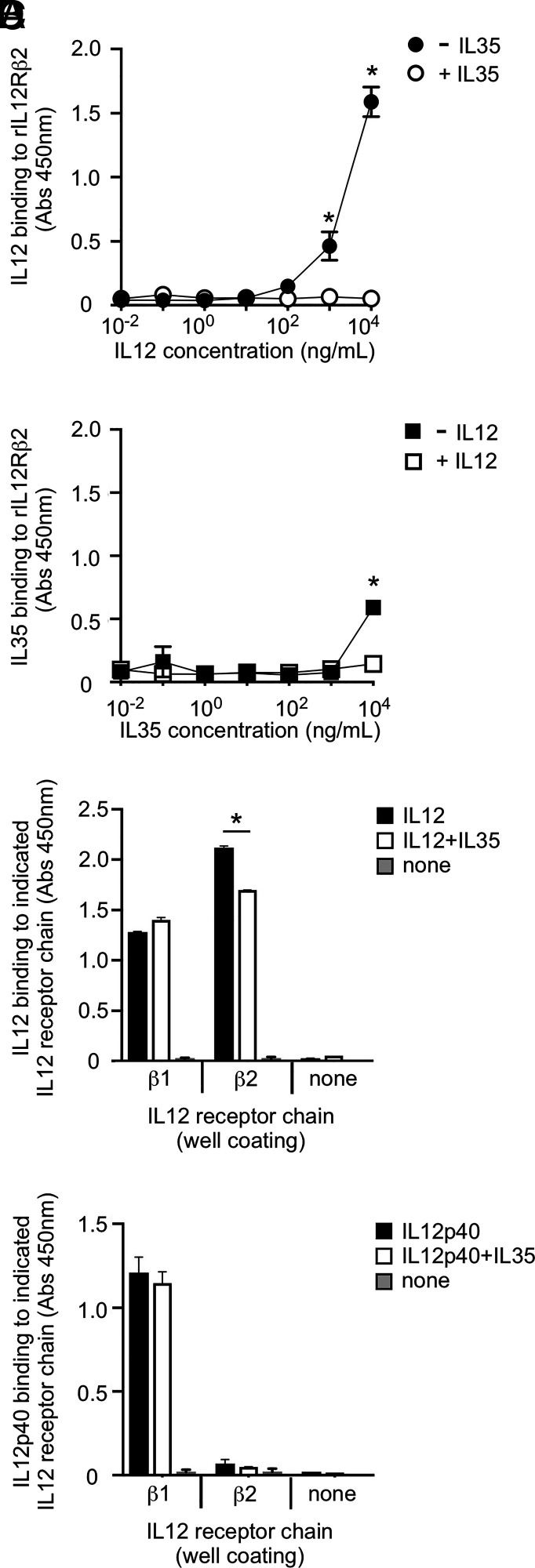
IL-35 and IL-12 antagonize the binding of one another to IL-12Rβ2. (**A**, **B**) Recombinant human IL-12Rβ2 (rIL-12Rβ2) was immobilized on wells of a sterile plate (high protein binding); each well was coated with the same total amount of rIL-12Rβ2. Immobilized rIL-12Rβ2 was subsequently overlaid with media containing (A) variable IL-12 concentrations with or without a fixed IL-35 concentration (10 μg/ml) or, alternatively, (B) variable IL-35 concentrations with or without a fixed IL-2 concentration (10 μg/ml). The plate was subsequently washed and stained with HRP-conjugated Abs specific to (A) IL-12 or (B) IL-35. HRP substrate was added, enabling quantitation of each cytokine’s binding to immobilized rIL12Rβ2. (**C**, **D**) In an analogous manner, wells were coated with either recombinant human IL-12Rβ1 (rIL-12Rβ1), rIL-12Rβ2, or no IL-12R chain (none). The following cytokines and treatments were then applied: (C) IL-12 alone, IL-12 and IL-35, or no cytokine at all, followed by washing and application of HRP-conjugated anti–IL-12; (D) IL-12p40 alone, IL-12p40 and IL-35, or no cytokine at all, followed by washing and application of HRP-conjugated anti–IL-12p40. In each case, the final concentration of each cytokine was fixed (5 μg/ml). After a final wash, HRP substrate was applied to enable quantitation of IL-12 and IL-12p40 binding to each IL-12R chain. Results are representative of three independent experiments. *p < 0.05 as determined by one-way ANOVA.

As an additional means of testing if human IL-35 prevents IL-12 from associating with IL-12Rβ2, we developed an ELISA-like solid-state binding assay wherein plate-bound recombinant human IL-12Rβ2 (rIL-12Rβ2) was overlaid with an aqueous solution comprising IL-12 at increasing concentrations with or without IL-35 at a single fixed concentration. After overnight incubation, the plate was washed to remove unbound cytokine and subsequently stained with anti–IL-12 Ab (HRP conjugated), enabling detection and quantitation of rIL-12Rβ2–bound IL-12. The results of this experiment are shown in Fig. 4A and demonstrate that the addition of IL-35 caused a significant decrease in IL-12 binding. We also performed the reciprocal experiment wherein plate-bound rIL-12Rβ2 was overlaid with an aqueous solution comprising IL-35 at increasing concentrations with or without IL-12 at a single fixed concentration. Plates were incubated overnight, washed, and otherwise treated in an identical manner, the only exception being that we instead used HRP-conjugated anti–IL-35 to detect and quantify rIL-12Rβ2–bound IL-35. The results of this experiment are shown in Fig. 4B and demonstrate that the addition of IL-12 caused a significant decrease in IL-35 binding.

To determine if IL-35 inhibition of IL-12 binding was specific to IL-12Rβ2 or if IL-12 binding to IL-12Rβ1 was also affected, we performed a similar experiment wherein wells were coated with either recombinant human IL-12Rβ1 (rIL-12Rβ1), rIL-12Rβ2, or no IL-12R chain at all. IL-12 was then applied with or without IL-35, followed by washing and application of anti–IL-12 detection Ab. As anticipated, IL-12 bound to both rIL-12Rβ1 and rIL-12Rβ2 (Fig. 4C); however, only IL-12 binding to rIL-12Rβ2 was negatively affected by the addition of IL-35 (Fig. 4C), suggesting that IL-12’s interaction with IL-12Rβ1 via its p40 domain is unaffected by IL-35. To further rule out the involvement of IL-12Rβ1, we repeated this experiment with p40 monomer. Consistent with prior literature (20, 21), p40 monomer binding was specific to rIL-12Rβ1, not rIL-12Rβ2 (Fig. 4D); the addition of IL-35 did not disrupt p40 monomer binding to IL-12Rβ1 (Fig. 4D). Collectively, these data support a model wherein IL-35 disrupts IL-12 binding to the IL-12Rβ2 component of its receptor, not IL-12Rβ1, and IL-12 likewise disrupts IL-35 binding to IL-12Rβ2.

### Human IL-35 can inhibit T cell responses to IL-12 in the absence of Treg or Breg cells

**FIGURE 5.**
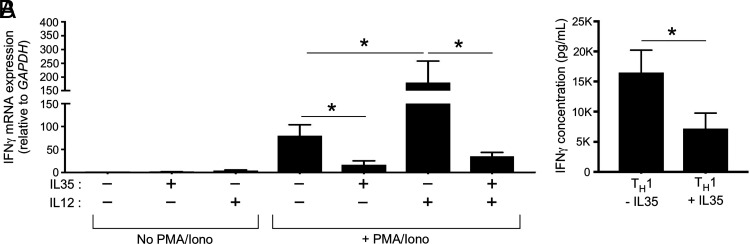
IL35 directly suppresses IL-12–elicited T cell expression of IFN-γ. (**A**) The Jurkat T cell line was cultured overnight in the absence or presence of PMA/Iono with or without IL-35 alone, IL-12 alone, or IL-12 and IL-35 together (IL-12 + IL-35). Cell lysates were collected and used to measure IFN-γ mRNA expression levels, as normalized to GAPDH. (**B**) Naive Th cells from the peripheral blood of healthy adult donors were purified and cultured in Th1 differentiation conditions (αCD3/αCD28 beads, IL-2, IL-12, anti–IL-4) with or without IL-35 for 1 wk. Each donor Th differentiation culture condition was set up in triplicate. At the end of this culture period, the cells in each culture condition were washed and stimulated with PMA/Iono. Shown are IFN-γ protein levels in the supernatants of stimulated cells. Results are representative of three independent experiments using blood from three separate donors. *p < 0.05 as determined by Student *t* test.

In the conventional model of IL-35 immunosuppression, Treg and Breg cells are cellular intermediates wherein IL-35 signaling via gp130/IL-12Rβ2 elicits their secretion of TGF-β1, which in turn suppresses Ag-specific T cells. We do not dispute this model; however, the above data suggest that IL-35 may also directly inhibit IL-12 binding to its receptor, regardless of whether Treg or Breg cells are present. To test if IL-35 directly inhibits IL-12–dependent T cell responses, independent of Treg and Breg cells, we used two model systems: the Jurkat T cell line (Fig. 5A) and naive Th cells from the peripheral blood of healthy adult donors (Fig. 5B). Jurkat T cells, which do not express the Treg transcription factor Foxp3 (22), were cultured overnight in the absence or presence of polyclonal stimulation (PMA/Iono) with or without IL-35 alone, IL-12 alone, or IL-12 and IL-35 (IL-12+IL-35). Jurkat expression of IFN-γ was used as a functional readout of IL-12 responsiveness. In the absence of PMA/Iono, IFN-γ mRNA levels were minimal, regardless of which cytokines were present (Fig. 5A). PMA/Iono elicited a significant increase in IFN-γ mRNA levels, which were suppressed in the presence of IL-35 (Fig. 5A). PMA/Iono and IL-12 together elicited the highest IFN-γ mRNA levels; however, in the presence of PMA/Iono and IL-12 + IL-35, IFN-γ mRNA levels declined to below PMA/Iono alone levels (i.e., the addition of IL-35 to the media negated IL-12’s positive effect on stimulated Jurkat IFN-γ mRNA expression) (Fig. 5A). We next assessed if the addition of IL-35 to naive Th cells cultured in Th1 differentiation conditions affected their subsequent production of IFN-γ. Naive peripheral blood Th cells were differentiated Th1 conditions with or without IL-35 for 1 wk, after which they were washed and restimulated with PMA/Iono. Consistent with an effect on IL-12 signaling, the presence of IL-35 during the Th1 differentiation process resulted in cells having a diminished capacity for IFN-γ secretion upon restimulation (Fig. 5B).

## Discussion

Despite their structural similarities, IL-12 and IL-35 have widely divergent effects on lymphocyte activity in disease model systems. The translational potential of IL-35 neutralization has been most explored in the context of cancer, because the anticancer activities of tumor-infiltrating lymphocytes are constrained by IL-35 in animal models of melanoma (5, 23) and pancreatic cancer (24), as is the therapeutic efficacy of PD1 blockade (25). In the tumor environment, IL-35 is produced by and signals through Treg and Breg cells to inhibit tumor-infiltrating lymphocyte activity (2). This conceptual model, wherein Treg and/or Breg cells are intermediates in the IL-35 immunosuppressive mechanisms, has been extended to a number of infectious diseases in which IL-35 levels or expression correspond to negative clinical outcomes, including viral hepatitis (26–29) and TB (30, 31). We do not dispute this model, but because IL-35 and IL-12 both have a p35 domain, we hypothesized that in addition to functioning through Treg and Breg cells, IL-35 may also compete with IL-12 for binding IL-12Rβ2, the transmembrane protein component of both the IL-12R and IL-35R that bind p35 (32). This is important to test because there are some tissues (e.g., the TB granuloma) wherein Treg and Breg cells are largely absent but in which IL-35 retains suppressive potential. Also, in the context of cancer, IL-12 has antiangiogenic properties that are being harnessed for experimental therapeutic purposes (33–35), which must overcome proangiogenic properties of IL-35 (23, 36) and cannot occur in the absence of IL-12Rβ2 binding (37). It is important, therefore, to have a comprehensive understanding of how IL-35 and IL-12 interact with one another.

The discovery of IL-35 stemmed in part from its expression in human placenta (38, 39), an immunosuppressive environment that protects the developing fetus from allorejection. Then referred to as “EBI3/p35 hematopoietin” (38), it was later termed IL-35 during investigations of its function in mouse models of IBD (4). Unlike IL-12, the subunits of which (IL-12p40 and IL-12p35) are primarily expressed by myeloid-derived APCs, the subunits of IL-35 (Ebi3 and IL-12p35) are expressed by APCs as well as regulatory lymphocyte subsets, signaling in the autocrine fashion described above, suppressing the T cell responses and cytokines that are pathogenic in the context of IBD and numerous other autoimmune disease models, including experimental autoimmune encephalomyelitis (EAE) (14). The mechanism our data support, wherein IL-35 prevents IL-12 from associating with IL-12Rβ2, is reminiscent of another IL-12 family member that suppresses lymphocyte activity: the IL-12p40 homodimer [IL-12(p40)_2_] (40). IL-12(p40)_2_ competes with IL-12 for access to the IL-12Rβ1 chain (20) and (similar to IL-35) suppresses IL-12–elicited IFN-γ secretion (41). IL-12p40 monomer also ameliorates EAE, albeit via a slightly different mechanism involving prevention of IL-12Rβ1 internalization (42).

Now, 25 years after its discovery (38, 39), IL-35 is known to affect and/or be secreted by numerous immune lineages, including T cells (43), B cells (44, 45), monocytes (46), macrophages (47), and DCs (48–50). Our own interest in IL-35 and its relationship to IL-12–driven processes stems from our having previously studied human IL-12R expression and the impact of IL-12 signaling on *Mycobacterium tuberculosis* resistance (51–53), as well as the excellent review of Ye et al. (14), who concluded it was important for future investigations to test the interaction of IL-35 and IL-12, given their shared receptor use. Given the positive correlation between IL-35 expression and active TB status (30, 31), we are now keenly interested in testing whether IL-35 suppresses *M. tuberculosis*–elicited Th1 cells via direct competition with IL-12 for IL-12Rβ2 or indirectly via the regulatory cells that slow the onset of adaptive immunity (54). Our present study uses human cell cultures and in vitro systems, so it cannot distinguish the relative importance of direct versus indirect suppression of Th1 cells by IL-35 in vivo, because this can only be addressed using animal models of diseases that are influenced by Th1 cells, such as TB (55) or the transfer colitis model (i.e., transferring naive T cells into lymphopenic mice, wherein Scurfy donor cells could be used to rule out effects on Treg cells) (56). With regard to TB, the limited mouse model data to date are certainly consistent with IL-35 suppressing host-protective adaptive immune responses: Mice injected with the avirulent *M. tuberculosis* strain H37Ra demonstrate that the highest lung IL-35 expression coincides with high bacterial burdens (57). Similar results are observed in mice i.v. injected with the vaccine strain *M. bovis* bacillus Calmette-Guérin (30); macrophages infected in vitro with the virulent *M. tuberculosis* strain H37Rv likewise accumulate Ebi3 protein (58). To our knowledge, the phenotype of virulent *M. tuberculosis*–infected Ebi3^−/−^ mice has not been reported (although these mice would lack both IL-35 and IL-27, which also uses the Ebi3 domain).

We found it curious that IL-35 most potently suppressed IL-12 bioactivity at lower IL-35 concentrations (IL-35^LO^) compared with higher IL-35 concentrations (IL-35^HI^). IL-35^LO^ and IL-35^HI^ both suppressed IL-12–elicited reporter activity (Fig. 1C–1F); unlike IL-35^LO^, however, IL-35^HI^ suppression was seen only at high IL-12 concentrations. Although these data are admittedly perplexing, they are not at odds with our proposed model of direct competition (Fig. 1A). First, and per the work of Collison et al. (15), IL-35 can induce higher-order assembly structures of IL-12Rβ2:IL-12Rβ2 homodimers that bind IL-35 and signal independently of gp130; so, too, however, can IL-12 induce IL-12Rβ2:IL-12Rβ2 homodimers (15) and signal independently of IL-12Rβ1, albeit not as robustly as when IL-12Rβ1 is present (59). In a setting of simultaneously high IL-12 and high IL-35 concentrations, these higher-order IL-12Rβ2 homodimer assemblies are likely forming and signaling, thus masking the suppression observed with lower IL-35 concentrations. Second, and per the early studies of IL-12R signaling done at Hoffman-LaRoche (reviewed in Ref. 16), IL-12Rβ1 oligomers (not monomers) bind IL-12 (60), and, although IL-12Rβ1, which is homologous to gp130, primarily binds to IL-12 via its IL-12p40 domain, it nevertheless makes contact with the IL-12p35 domain per structural modeling studies (61). In IL-35^HI^ conditions, the IL-12p35 domain of IL-35 may be interacting with the IL-12Rβ1 oligomers that surround IL-12, triggering the activity of their associated intracellular kinases (62).

Collison et al. (15) were first to demonstrate IL-12Rβ2’s involvement in IL-35 signaling, because the application of exogenous IL-35 brings IL-12Rβ2 into physical proximity with gp130 (sufficiently close for fluorescence resonance energy transfer excitation), and IL-12Rβ2–deficient mouse T cells are partially resistant to IL-35–mediated suppression. Our data are consistent with the work of Collison et al. (15) insomuch as we observed human IL-12Rβ2 to coimmunoprecipitate with IL-35 (Fig. 2F). Nevertheless, because direct evidence of IL-35 binding to the human IL-12Rβ2 receptor is missing in terms of a binding constant (such as competitive binding using a radiolabeled ligand) or from biophysical measurements such as surface plasmon resonance spectroscopy or isothermal titration calorimetry, and because a crystal structure of human IL-12Rβ2 in complex with IL-35 has not been published, we cannot rule out the possibility that, in our system, IL-35 may also be suppressing IL-12 bioactivity via nonspecific interactions such as binding to cell surface proteoglycans.

To conclude, our data support a mechanism wherein human IL-35 suppresses IL-12 signaling via competition for IL-12Rβ2 binding, which associates with the p35 domain of both cytokines. A limitation of our work may be its having been performed primarily using cell lines, recombinant proteins, and solid-state binding assays (as opposed to mice); however, because there are substantial differences between mouse and human p35 at the amino sequence level (17) and activity level, and because human IL-12 does not act on mouse cells, whereas mouse IL-12 acts on both human and mouse cells (18), we believe the experimental systems we chose were ideal to ensure relevance to human immune responses.

## Supplementary Material

Supplemental Figures 1 (PDF)
