# Supplementary material for: Human IL-35 Inhibits the Bioactivity of IL-12 and Its Interaction with IL-12Rβ2
Source: Immunohorizons. 2023 Jun 8;7(6):431–41. doi: 10.4049/immunohorizons.2300039 (PMC10580122; doi:10.4049/immunohorizons.2300039)
Supplement: Supplemental Figures 1 (PDF) [file IH_2300039_Supplemental_1.pdf]

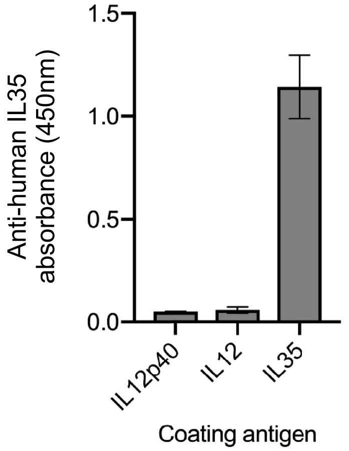

Supplemental FIG S1. The anti-human IL35 antibody used in our studies does not cross-react with human IL12. To validate the anti-IL35 antibody purchased for our studies, individual wells of a 96-well plate were coated overnight with either IL12p40 monomer, IL12, or IL35. Wells were then washed (5X with PBS-Tween), treated with HRP-conjugated mouse anti-human IL35 antibody (1 hr at room temp), washed again and HRP substrate applied. Shown are the absorbance values of wells coated with each cytokine, with each bar representing the mean ±SD.

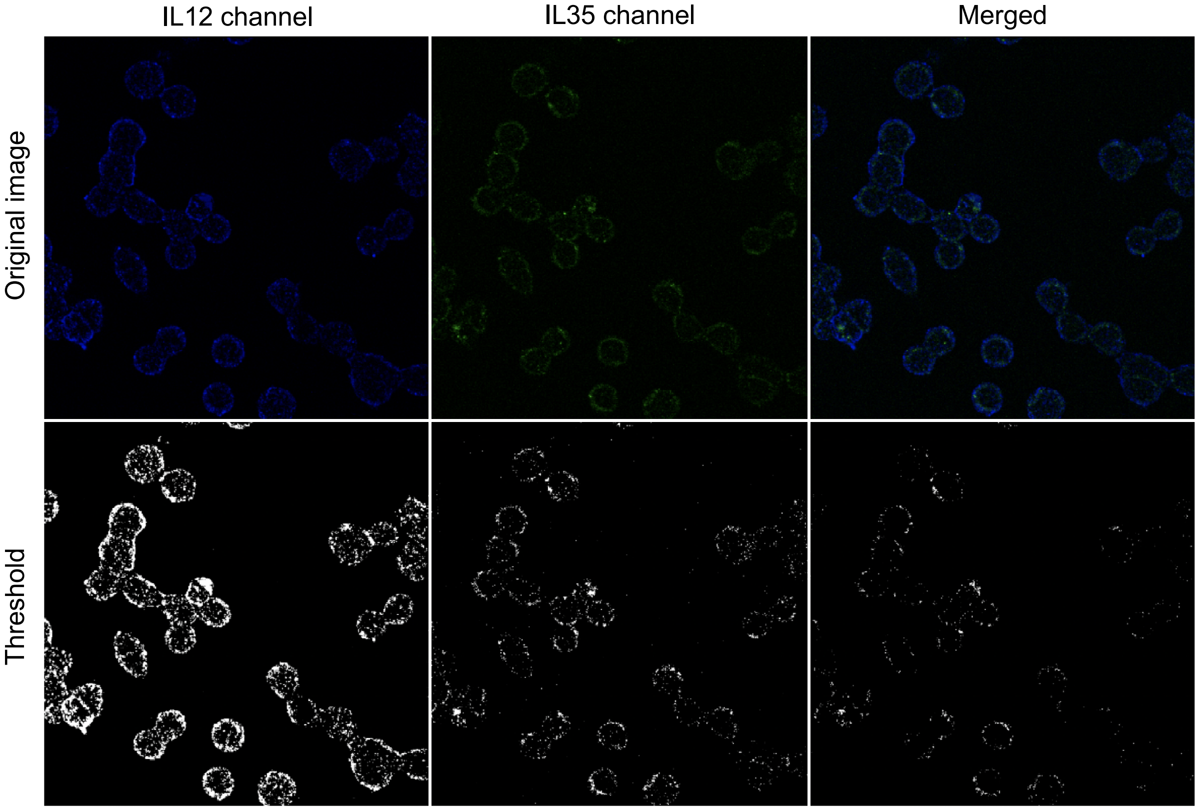

Supplemental FIG S2. Quantification of IL12 and IL35 colocalization. For colocalization analysis of IL12 and IL35 on the surface of reporter cells, images of cells that had been treated with both cytokines and subsequently stained with fluorophore-conjugated anti-IL12 and anti-IL35 (i.e. those shown in **FIG 2C**) were analyzed using FIJI. The top panel are the original immunofluorescence images of the anti-IL12 fluorescence channel (left row), anti-IL35 fluorescence channel (middle row), and merged channels (right row); the bottom panel are the corresponding manual thresholds used to generate the 2D intensity fluorogram shown in **FIG 2E**. The threshold values chosen for the IL12 and IL35 channels were 50 and 40, respectively.
